# Procalcitonin as a Biomarker for Malignant Cerebral Edema in Massive Cerebral Infarction

**DOI:** 10.1038/s41598-018-19267-4

**Published:** 2018-01-17

**Authors:** Yan Zhang, Gang Liu, Yuan Wang, Yingying Su, Rehana K. Leak, Guodong Cao

**Affiliations:** 1 Department of Neurology, Xuanwu Hospital, Capital Medical University, Beijing, 100053 China; 2 Division of Pharmaceutical Sciences, Duquesne University, Pittsburgh, PA 15282 USA; 3 Department of Neurology, University of Pittsburgh, Pittsburgh, PA 15260 USA; 4 Geriatric Research Education and Clinical Centers, VA Pittsburgh Healthcare System, Pittsburgh, PA 15240 USA

## Abstract

The objective of this study is to explore whether procalcitonin (PCT) can serve as an early biomarker of malignant cerebral edema in patients with massive cerebral infarction (MCI). Ninety-three patients with acute MCI were divided into death or survival groups based on whether they died or survived within 1 week of cerebral herniation. Differences in laboratory parameters between these two groups were analyzed by univariate analysis, followed by multivariate logistic regression analyses if the influencing factors were significantly different. Compared with the survival group, the patients in the death group had a larger cerebral infarct area, higher body temperature, neutrophil counts, PCT level, and neuron-specific enolase (NSE) level within 48 h of onset. Multivariate logistic regression analyses revealed an odds ratio (OR) of 1.830 or 1.235 for PCT and neutrophil counts respectively, suggesting that PCT and neutrophil counts are two independent risk factors for death in MCI. The area under receiver operating characteristic (ROC) curve was 0.754 for PCT, larger than that for neutrophil counts. Thus, both serum PCT levels and neutrophil counts can be used as biomarkers to predict malignant cerebral edema at the early stages after MCI, but PCT levels are superior predictors of malignant cerebral edema.

## Introduction

The one-year mortality rate for massive MCI in the middle cerebral artery (MCA) territory is as high as 71–80%, and most patients die from cerebral herniation caused by malignant cerebral edema within one week^1,2^. At present, the pathogenesis of malignant cerebral edema is not completely understood. It is difficult to reverse the occurrence of cerebral herniation with medication alone, although decompressive craniectomy can significantly reduce the mortality^3^. Therefore, the early prediction of malignant cerebral edema in patients with MCI will facilitate the timely selection of appropriate treatment regimens.

The inflammatory response is an important factor in ischemic stroke^4,5^, and inflammatory markers such as PCT and high-sensitivity C-reactive protein (CRP) can help to determine the severity and prognosis of acute cerebral infarction^6–10^. PCT has been widely used in clinical practice as a diagnostic marker for serious bacterial infections and sepsis, as well as a prognostic marker for patients with infections^11,12^. Recent studies have found that PCT is an independent risk factor for cerebral infarction^7,13,14^. Studies have also identified serum PCT as an independent risk factor for poor prognosis and mortality with better prediction than CRP^15,16^. However, whether PCT could be used as a biomarker for malignant cerebral edema following MCI has not been investigated. Therefore, this prospective study evaluated whether PCT can serve as a biomarker to improve the early diagnosis and guide the surgical treatments for malignant cerebral edema in patients with MCI.

## Methods

### Inclusion and exclusion criteria of patients

This study enrolled patients with acute MCI admitted to the neuro-intensive care unit (NCU) of Xuanwu Hospital in Capital Medical University from January 2011 to June 2016. This study was approved by the Ethics Committee of Xuanwu Hospital, Capital Medical University. Informed consent was obtained from patients or their guardians. The diagnosis and management of MCI were carried out in accordance with the guidelines for the management of large hemispheric infarction^17^. The inclusion criteria were as follows: (1) Unilateral MCI involving at least 2/3 of the MCA territory, as confirmed by head computed tomography (CT) or magnetic resonance imaging (MRI); (2) within 48 h of MCI onset. The exclusion criteria were as follows: (1) Treatment with thrombolytic or surgical treatments; (2) MCI with intracranial hematomas; and (3) death due to serious systemic diseases, including multiple organ failure and severe infections. In addition, we enrolled only MCI patients who were excluded from decompressive surgery for the following reasons: 1) patients taking antiplatelet drugs were excluded from decompressive surgery to avoid postoperative bleeding; 2) patients without obvious unconsciousness were excluded; 3) some patients were excluded due to patients’ or guardians’ concerns about long-term disability after surgery; and 4) patients with financial concerns were excluded. The enrollment of MCI patients who were excluded from decompressive surgery allowed us to investigate the role of MCI and inflammatory markers without the involvement of surgical factors. All patients were treated for brain edema with osmotic medications—either intravenous 20% mannitol or 10% hypertonic saline.

Patients were divided into the death group or survival group based on whether patients died from cerebral herniation or survived within one week of onset. Cerebral herniation was defined as the occurrence of certain clinical manifestations and the imaging examination (cranial CT or MRI), including severe cerebral edema, cerebral ventricular compression, and a midline shift.

### Clinical assessment and data collection

Blood samples were collected with or without anticoagulant at 48 h within MCI onset to test blood parameters such as white blood cell (WBC) counts, neutrophil counts, neutrophil ratios, and platelet counts, or to measure blood chemical/factors such as the levels of PCT, CRP, NSE, blood glucose, and blood sodium. All patients were administered clinical and imaging examinations within 48 h of disease onset, and the following information was recorded: (1) age; (2) sex; (3) history of smoking and drinking; (4) occurrence of headache, vomiting, disturbance of consciousness, and gaze palsy; (5) body temperature, blood pressure, and NIHSS score; (6) cerebral infarction location and range (shown by cranial CT or MRI), according to which the MCI was categorized as one of two types—infarcts involving only the area supplied by the MCA and those involving the region exceeding the MCA territory (involving the area supplied by the anterior cerebral artery or posterior cerebral artery); (7) etiology of cerebral infarction according to the TOAST classification (large-artery atherosclerosis, cardiogenic cerebral embolism, small-artery occlusion, other determined, and undetermined etiology)^18^; and (8) whether accompanied by atrial fibrillation, cardiac dysfunction, abnormal liver function, abnormal renal function, or infection.

### Statistical analysis

Statistical analyses were performed with the statistical software SPSS 22.0 (IBM Corporation, Armonk, NY). Measurement data with normal distributions were expressed as the mean ± standard deviation, while data with non-normal distributions were expressed as the median (interquartile range, IQR). The Student’s *t* test was used for intergroup comparisons of measurement data with a normal distribution and homogeneous variance, while the Mann-Whitney U test was employed for intergroup comparisons of measurement data with a non-normal distribution and heterogeneous variance. Binary measurement data were analyzed using Fisher’s exact test. Univariate analyses were employed to examine the differences in each observed indicator between the death and survival groups, and the influencing factors that were significantly different were further analyzed using multivariate logistic regression analyses. The ROC curve, a parameter to reflect the sensitivity and specificity of continuous variables, was used to evaluate the diagnostic performance of the independent risk factors for death. The differences of the area under the ROC curve were deemed statistically significant only when P ≤ 0.05.

## Results

**Figure 1:**
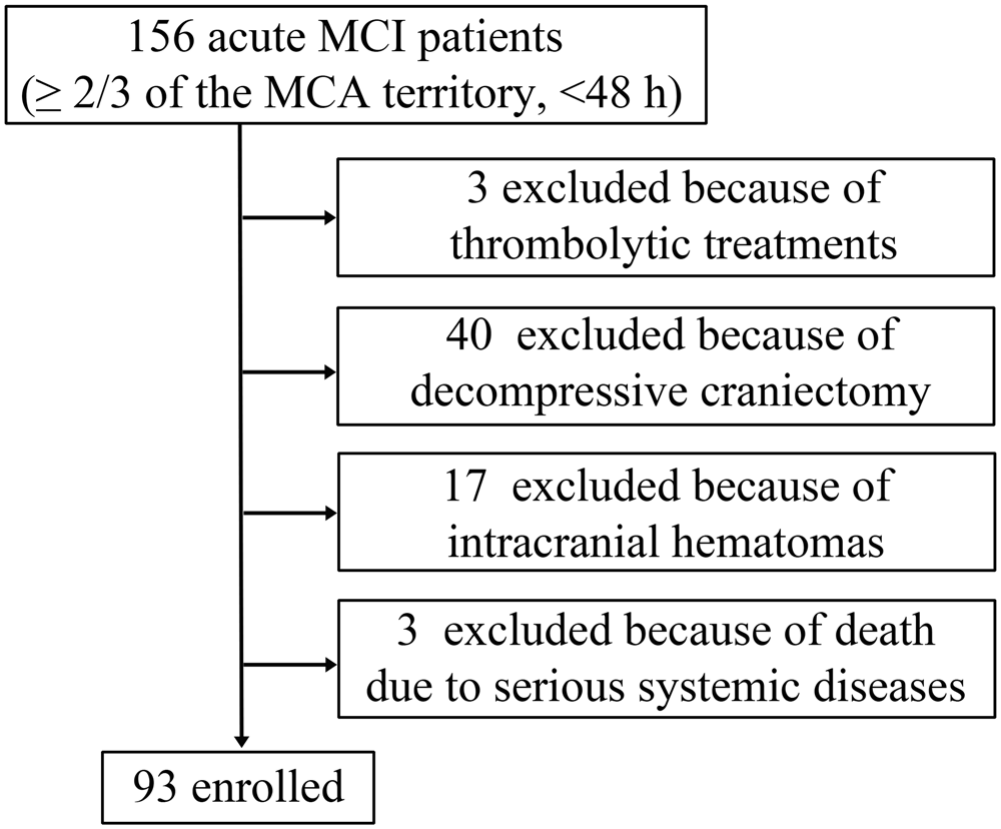
Patient enrollment flow chart.

As shown in the patient enrollment flow chart in Fig. 1, a total of 93 patients, including 63 males and 30 females (mean age: 62.8 ± 13.4 years old) were enrolled. There were 25 patients in the death group and 68 patients in the survival group. The univariate analysis indicated no differences between the two groups (P > 0.05) in age, sex, smoking, and drinking history, incidence of headache, vomiting, disturbance of consciousness and gaze palsy, blood pressure, National Institutes of Health Stroke Scale (NIHSS) score, TOAST classification, WBC count, platelet count, CRP, blood glucose, blood sodium, concomitant atrial fibrillation, cardiac dysfunction, abnormal liver function, abnormal renal function, infection within 48 h of onset, and anti-edema therapy. However, compared with the survival group, the patients in the death group had a larger cerebral infarct area, a higher body temperature (37.18 ± 0.82 °C *vs*. 36.81 ± 0.50 °C), higher neutrophil counts [(11.15 ± 4.21) × 10^9^/L *vs*. (9.41 ± 3.16) × 10^9^/L], higher PCT levels [0.259 (interquartile range of 0.084–5.000) ng/mL *vs*. 0.069 (interquartile range of 0.042–0.154) ng/mL], and higher NSE levels [32.00 (interquartile range of 20.51–62.72) ng/mL *vs*. 22.20 (interquartile range of 17.52–38.78) ng/mL] within 48 h of onset (P < 0.05) (Table 1).

**Table 1:** Demographic and clinical differences between dead and surviving patients with MCI.

	Survival (n = 68)	Dead (n = 25)	P value
Age (years), mean ± SD	62.7 ± 14.4	62.9 ± 10.4	0.953
Male sex, n (%)	48 (70.6)	15 (60.0)	0.453
Smoking, n (%)	41 (60.3)	11 (44.0)	0.239
Alcohol, n (%)	34 (50.0)	9 (36.0)	0.251
Vomiting, n (%)	17 (25.0)	9 (36.0)	0.308
Headache, n (%)	13 (19.1)	4 (16.0)	1.000
Disorder of consciousness, n (%)	45 (66.2)	20 (80.0)	0.308
Paralysis of gaze, n (%)	38 (55.9)	13 (52.0)	0.816
Vascular territory involvement			0.020
MCA territory, n (%)	58 (85.3)	15 (60.0)	
Exceeding the MCA territory, n (%)	10 (14.7)	10 (40.0)	
Stroke etiology			0.540
Large-vessel occlusive, n (%)	42 (61.8)	17 (68.0)	
Cardioembolic, n (%)	23 (33.8)	8 (32.0)	
Others, n (%)	3 (4.4)	0 (0)	
Body temperature (°C), mean ± SD	36.81 ± 0.50	37.18 ± 0.82	0.011
Systolic blood pressure (mmHg), mean ± SD	158.4 ± 22.0	155.3 ± 24.7	0.565
Diastolic blood pressure (mmHg), mean ± SD	86.1 ± 14.6	83.4 ± 15.8	0.439
NIHSS, mean ± SD	17.6 ± 5.8	19.7 ± 6.7	0.147
WBC counts (×10^9^/L), mean ± SD	11.64 ± 3.59	11.99 ± 3.90	0.684
Neutrophil counts (×10^9^/L), mean ± SD	9.41 ± 3.16	11.15 ± 4.21	0.035
Neutrophil (%), median (IQR)	84.9 (77.6–88.6)	83.8 (77.4–90.9)	0.649
CRP (mg/L), median (IQR)	27.00 (8.64–70.90)	34.20 (15.45–68.05)	0.309
PCT (ng/L), median (IQR)	0.069 (0.040–0.150)	0.259 (0.084–5.000)	<0.001
NSE (ng/L), median (IQR)	22.02 (17.52–38.78)	32.00 (20.51–62.72)	0.049
Platelet counts (×10^9^/L), mean ± SD	206.2 ± 70.2	189.3 ± 61.2	0.292
Glucose (mmol/L), median (IQR)	7.39 (5.90–9.78)	8.00 (6.59–10.54)	0.398
Serum sodium(mmol/L), mean ± SD	139.58 ± 4.09	140.43 ± 6.75	0.462
Atrial fibrillation, n (%)	17 (25.0)	7 (28.0)	0.793
Heart failure, n (%)	18 (26.5)	6 (24.0)	0.308
Abnormal liver function, n (%)	20 (29.4)	6 (24.0)	0.795
Abnormal renal function, n (%)	8 (11.8)	5 (20.0)	0.325
Infection, n (%)	54 (79.4)	20 (80.0)	1.000
Osmotic medications			0.308
20% mannitol, n (%)	51 (75.0)	16 (64.0)	
10% hypertonic saline, n (%)	17 (25.0)	9 (36.0)	

**Figure 2:**
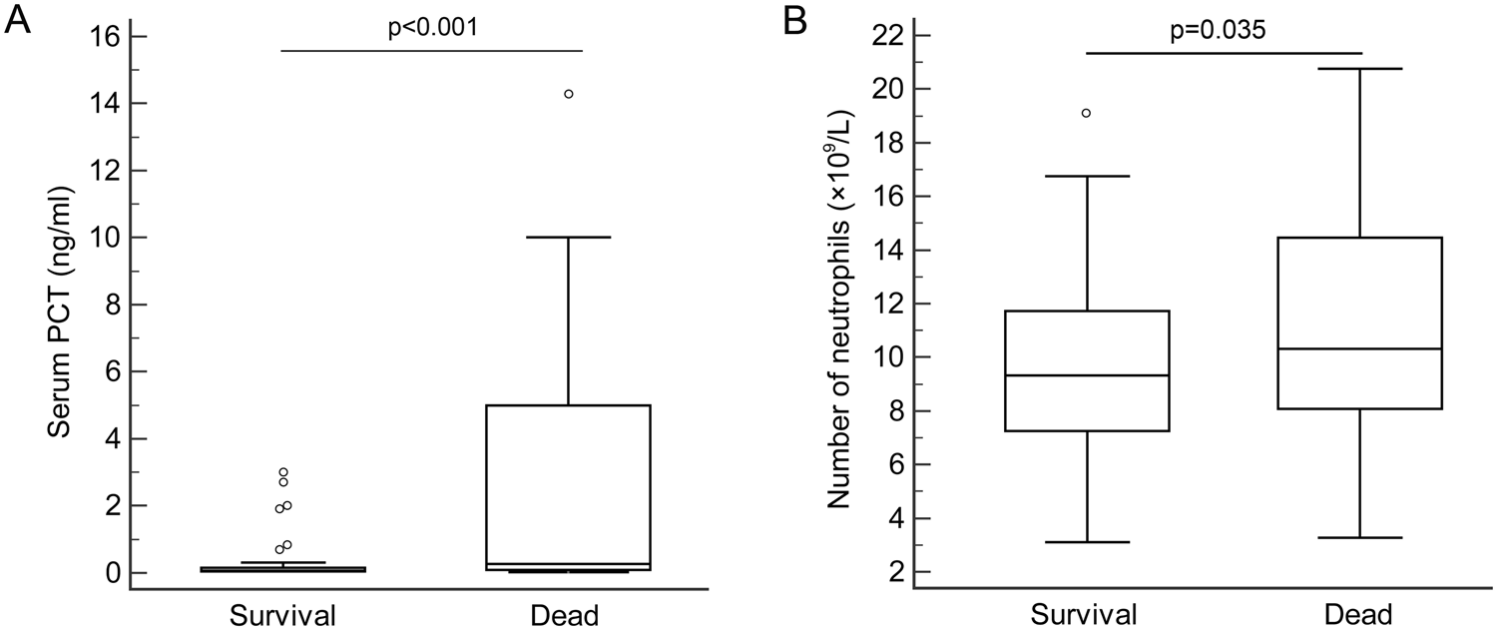
Distributions of serum levels of PCT and neutrophil counts in survivors and non-survivors. The range of variation of serum PCT levels (**A**) and neutrophil counts (**B**) is displayed with box plots. IQR is denoted with an open box; maximum, minimum, and median values are denoted by top, bottom, and middle lines, respectively. Outliers are denoted by circles. P < 0.001 or =0.035 dead versus survival, n = 68 for survivors and n = 25 for non-survivors.

The distribution of serum PCT and neutrophil counts was displayed with box plots. As shown in Fig. 2A, the univariate analysis indicated that patients in the death group had significantly higher PCT levels (median = 0.259 ng/mL) compared with the survival group (median = 0.069 ng/mL) (P < 0.001). Similarly, the analysis of neutrophil counts (Fig. 2B) showed that the death group had higher neutrophil counts compared with the survival group (median = (11.15 ± 4.21) × 10^9^/L and (9.41 ± 3.16) × 10^9^/L, respectively, p = 0.035).

To further investigate whether the infarct volume, body temperature, neutrophil counts, PCT levels, and NSE levels could be used as biomarkers or independent risk factors for malignant cerebral edema, we performed multivariate logistic regression. The results indicated significant differences in PCT levels (P = 0.005) and neutrophil counts (P = 0.018) between the death and survival groups within 48 h of onset (Table 2). After correction for infarct range, body temperature, and NSE levels, PCT and neutrophil counts were shown to be independent risk factors for death due to cerebral herniation with an OR of 1.830 (95% confidence interval of 1.197–2.797) and 1.235 (95% confidence interval of 1.037–1.470), respectively.

**Table 2:** Multivariate logistic regression analyses of early death in patients with massive cerebral infarction.

Predictors	OR	95% CI	P value
PCT	1.830	1.197–2.797	0.005
Neutrophil counts	1.235	1.037–1.470	0.018
Vascular territory involvement	2.997	0.830–10.822	0.094
Body temperature	2.156	0.824–5.592	0.118
NSE	1.012	0.993–1.032	0.205

**Figure 3:**
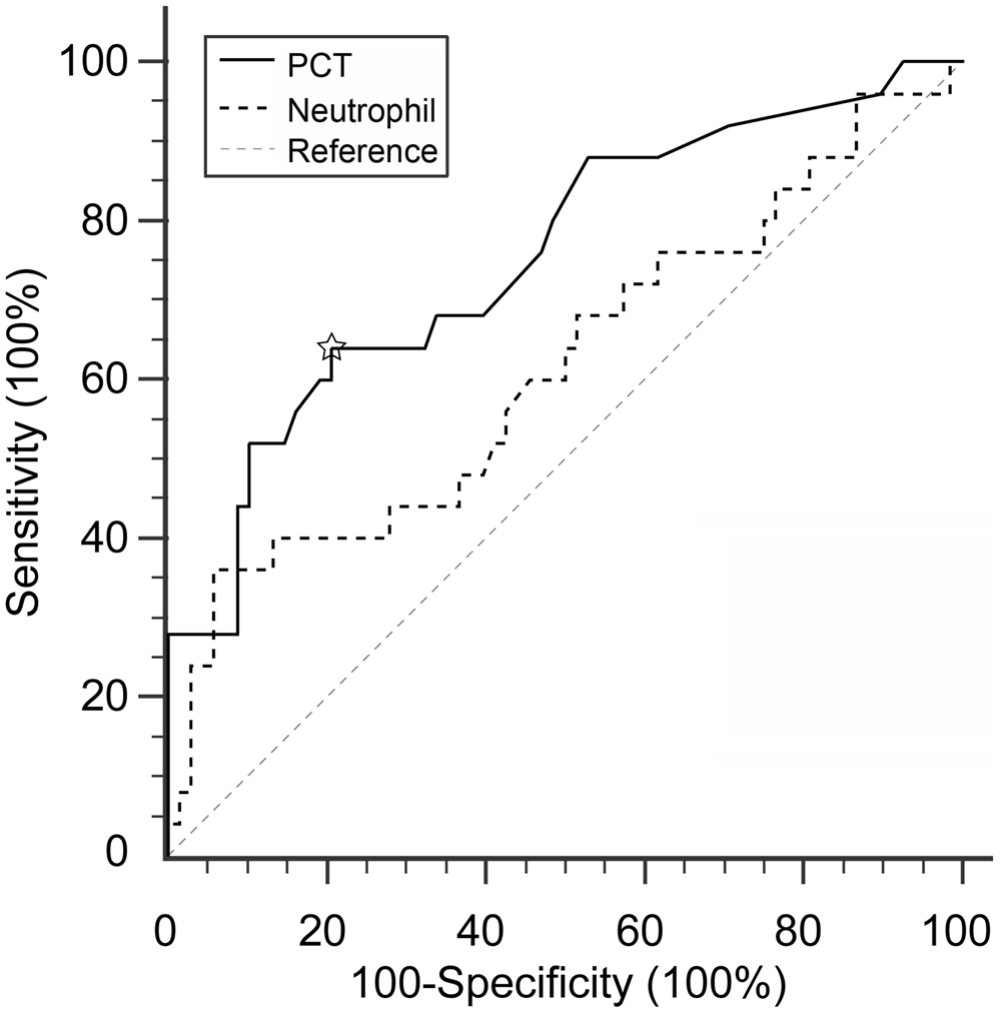
The ROC curves of serum PCT levels and neutrophil counts. The reference line is denoted as the dotted diagonal. ^☆^The optimal cutoff value of PCT level.

The ROC curve was utilized to evaluate the accuracy of serum PCT levels and neutrophil counts as risk factors that predict death caused by cerebral herniation. As shown in Fig. 3, the optimal cutoff value for serum PCT as a risk factor was 0.18 ng/ml. The sensitivity of the cutoff value was 64% (95% confidence interval of 42.5–82.0%), and its specificity was 79.4% (95% confidence interval of 67.9–88.3%). The area under the ROC curve was 0.754 (95% confidence interval: 0.653–0.837), which was larger than that of the neutrophil counts curve (0.616; 95% confidence interval: 0.509–0.715), suggesting that the PCT levels were superior biomarkers of early death in MCI patients.

## Discussion

The results of this study demonstrate that serum PCT levels and neutrophil counts are independent risk factors for early death due to malignant cerebral edema in patients with MCIs. Compared with neutrophil counts, PCT levels were superior biomarkers to predict early death in MCI patients. Serum PCT has an optimal cutoff value of 0.18 ng/mL for predicting death due to cerebral herniation in MCI patients, with a sensitivity of 64.0% and a specificity of 79.4%. These data suggest that the serum PCT level not only is correlated with the incidence of cerebral infarction and its severity but also plays a role in the pathological progression of malignant cerebral edema associated with MCI.

PCT, the precursor of calcitonin, is generated at low levels in serum by thyroid C cells under physiological conditions. However, under pathological conditions such as bacterial infections, PCT levels in serum rise significantly^11,12^ and tissues other than the thyroid, such as the lungs and intestines, mainly produce PCT. PCT levels increase in cerebral infarction^7,13,14^ as well as in severe trauma^19,20^, even without bacterial infection^19,20^. Our study indicates that acute MCI significantly increases PCT levels in serum, consistent with previous reports^9^. It has been reported that the reduction of miR-637 in intestinal neuroendocrine cells leads to the release of more PCT into the bloodstream during acute cerebral infarction^21^.

Our study indicates that PCT is an independent risk factor for death caused by cerebral herniation due to an MCI. Previous studies of acute cerebral infarction found that serum PCT was an independent risk factor for poor prognosis and mortality, with better predictive performance than other known inflammatory markers, such as WBC counts, neutrophil counts, and CRP^9,15,16^. Studies of therapeutic hypothermia following cardiopulmonary resuscitation have also identified an increase in PCT as a strong predictor for poor prognosis of neurological functions, while CRP and WBC counts were shown to have no correlation with the prognosis of neurological functions^22^.

Although there were no statistically significant differences in WBC counts between the death and survival groups, our study indicates that neutrophil count is an independent risk factor for death from malignant cerebral edema in patients with MCI. A previous study has shown that leukocytosis was a risk factor for malignant cerebral edema in MCI^23^, but the study did not classify the WBC components for comparison. A meta-analysis indicated that neutrophil counts were correlated with myocardial infarction and ischemic stroke, and showed a stronger correlation with these two diseases than the other leukocyte components. In addition, this correlation was shown to be independent of the other risk factors for cardiovascular and cerebrovascular diseases, such as smoking^24,25^. Thus, the inflammatory process reflected by neutrophil counts has a closer correlation with ischemic stroke and malignant cerebral edema secondary to MCI.

The National Institutes of Health defines a biomarker as “a characteristic that is objectively measured and evaluated as an indicator of normal biological processes, pathogenic processes, or pharmacologic responses to a therapeutic intervention”^26^. Similarly, the World Health Organization defines a biomarker as “any substance, structure, or process that can be measured in the body or its products and influence or predict the incidence of outcome or disease”^27^. As PCT levels fit these criteria, we propose that PCT measurements can serve as a viable biomarker of malignant cerebral edema. However, one limitation of the current study is that PCT levels were only measured 48 h within MCI onset. PCT levels might change dynamically at stroke onset, at the peak of edema, or during the stroke recovery period, which warrants further study. Similar to traumatic brain injury, which is associated with high levels of PCT^19,20^, the decompressive craniectomy itself is an injury to the brain which may trigger inflammation and increase PCT levels. However, decompressive craniectomy can also reduce intracranial pressure/brain edema and attenuate the inflammatory response, thereby perhaps decreasing the level of inflammatory markers such as PCT. Thus, it will be important to analyze dynamic changes in PCT after decompressive craniectomy to determine whether PCT levels could be used as prognostic indicators in the context of MCI. This is one of the limitations of the current study, and it warrants further investigation.

In summary, serum PCT levels and neutrophil counts during the early stage of MCI can be used as biomarkers for malignant cerebral edema in MCI, with PCT levels being superior to neutrophil counts. The inflammatory response may play a role in the pathogenesis of malignant cerebral edema during MCI, but its mechanism requires further study.
